# Determining the post-elimination level of vaccination needed to prevent re-establishment of dog rabies

**DOI:** 10.1371/journal.pntd.0007869

**Published:** 2019-12-02

**Authors:** Seonghye Jeon, Julie Cleaton, Martin I. Meltzer, Emily B. Kahn, Emily G. Pieracci, Jesse D. Blanton, Ryan Wallace

**Affiliations:** 1 Emergency Preparedness and Response Branch, Division of Preparedness and Emerging Infections, National Center for Emerging & Zoonotic Infectious Diseases, Centers for Disease Control and Prevention, Atlanta, Georgia, United States of America; 2 Poxvirus and Rabies Branch, Division of High-Consequence Pathogens and Pathology, National Center for Emerging & Zoonotic Infectious Diseases, Centers for Disease Control and Prevention, Atlanta, Georgia, United States of America; US Department of Agriculture, UNITED STATES

## Abstract

**Background:**

Once a canine rabies-free status has been achieved, there is little guidance available on vaccination standards to maintain that status. In areas with risk of reintroduction, it may be practical to continue vaccinating portions of susceptible dogs to prevent re-establishment of canine rabies.

**Methods:**

We used a modified version of RabiesEcon, a deterministic mathematical model, to evaluate the potential impacts and cost-effectiveness of preventing the reintroduction of canine rabies through proactive dog vaccination. We analyzed four scenarios to simulate varying risk levels involving the reintroduction of canine rabies into an area where it is no longer present. In a sensitivity analysis, we examined the influences of reintroduction frequency and intensity, the density of susceptible dog population, dog birth rate, dog life expectancy, vaccine efficacy, rate of loss of vaccine immunity, and the basic reproduction number (R_0_).

**Results:**

To prevent the re-establishment of canine rabies, it is necessary to vaccinate 38% to 56% of free-roaming dogs that have no immunity to rabies. These coverage levels were most sensitive to adjustments in R_0_ followed by the vaccine efficacy and the rate of loss of vaccine immunity. Among the various preventive vaccination strategies, it was most cost-effective to continue dog vaccination at the minimum coverage required, with the average cost per human death averted ranging from $257 to $398 USD.

**Conclusions:**

Without strong surveillance systems, rabies-free countries are vulnerable to becoming endemic when incursions happen. To prevent this, it may be necessary to vaccinate at least 38% to 56% of the susceptible dog population depending on the risk of reintroduction and transmission dynamics.

## Author summary

Canine rabies is a fatal yet preventable disease. Once a canine rabies-free status has been achieved, there is little guidance available on vaccination standards to maintain that status. However, the risk of reintroduction and the associated costs may not be negligible. We used a modified version of RabiesEcon, a deterministic mathematical model, to evaluate the potential impacts and cost-effectiveness of preventing the reintroduction of canine rabies through a proactive dog vaccination. We found that vaccinating 38% to 56% of the susceptible dog population (i.e., free-roaming dogs with no immunity to rabies) could prevent the re-establishment of canine rabies within the first 2 years of the incursion. Among the various preventive vaccination strategies, it was most cost-effective to continue dog vaccination at the minimum coverage required, with the average cost per human death averted ranging from $257 to $398 USD. To maintain rabies-free status and protect the significant investments made to eliminate canine rabies, it may be necessary to vaccinate portions of susceptible dog populations depending on the risk of reintroduction, surveillance capacity, and transmission dynamics.

## Introduction

Globally there are approximately 59,000 annual human rabies deaths, with 98% of those deaths due to canine rabies virus variant (CRVV) [1, 2]. While most countries in the Americas and Europe have eliminated rabies from their dog populations, canine rabies is still endemic in 122 countries. Dog vaccination is the most effective method for eliminating canine rabies and the associated human deaths [3]. As a guideline, the World Health Organization (WHO) recommends that canine rabies endemic countries vaccinate 70% of their susceptible dogs each year for a minimum of 5–7 years in order to end transmission [4, 5]. The WHO declares a country free of risk for dog rabies if there is no indigenously acquired infection in humans or any animal species due to a dog rabies virus during the previous 2 years [4].

After a country is declared free from canine rabies, however, there is a risk that rabies can be reintroduced due to an importation (human-mediated introduction of an animal into the country), incursion (natural movement of the virus from an endemic border area into the free zone), or host shift from an enzootic rabies virus variant (e.g., bat to dog). In the United States, around 100,000 animals per year are tested for rabies at more than 100 labs, and reporting these results from the state to the national level is required [6]. Although domestic dogs are no longer considered a rabies reservoir, around 60 rabid dogs have been reported each year in the US during 2008–2017. These mostly become infected from other wildlife species such as bats, raccoons, and skunks and pose the potential risk of a host-shift of the virus back into the dog population [7]. In Europe and the Americas, rabies-free countries occasionally report the importation of rabid dogs and cats from endemic countries; during 2000–2013, 21 such incidents were recorded in Europe [8] and there have been 4 CRVV importation events in the United States during 2007–2017 [9].

Unfortunately, in recent years there are several examples of a reintroduction of the CRVV into previously free areas that initiated onward transmission. The city of Arequipa, Peru was declared free of rabies in the 1990s. In 2015, a canine rabies incursion from neighboring Bolivia occurred in Puno, a bordering region in Peru, then to the city of Arequipa. The Peruvian government has conducted a series of mass dog vaccination campaigns in an attempt to control the outbreak, but canine rabies virus transmission still continues in both cities [10]. The state of Sarawak in Malaysia had been free of rabies since 1999. However, the state announced a rabies outbreak in July 2017 after confirming two cases of rabies in children [11]. In spite of ring vaccination and animal control efforts, the rabies outbreak in Malaysia has spread throughout Sarawak and mainland Malaysia; and there are 19 rabies-related human deaths reported as of June 2019.

Historically, canine rabies introductions or reintroductions have been detected within a year in areas where rabies was eliminated or in islands with no history of rabies [12]. However, some of these events have still resulted in endemicity [12]. While a surveillance system may become strong while working to eliminate rabies, that system must be maintained long-term to continue detecting potential reintroductions. Ideally, countries will continue to invest in surveillance, but priorities sometimes shift when rabies is no longer causing human cases. In estimating the global burden of endemic canine rabies, Hampson et al. [2] did not consider mortality and costs due to imported cases in rabies-free countries. Nevertheless, as shown in recent cases from Peru and Malaysia, the risk of reintroduction and the associated costs may not be negligible.

For any country that has or will eliminate canine rabies, it is important to consider post-elimination control strategies to prevent re-establishment of endemic dog rabies due to a reintroduction. There are several post-elimination strategies such as border controls, surveillance, preventive and reactive vaccination. In this paper, we explored one of the options, a preventive vaccination strategy. While it is likely not practical or cost-effective to continue dog rabies vaccination at a level of 70% coverage, it is unknown what level of vaccination is necessary to prevent re-establishment of canine rabies. We estimated the minimum vaccination coverage required to prevent re-establishment of canine rabies if it is reintroduced into a canine rabies-free population. We also compared the cost-effectiveness of various vaccination scenarios. This information and the tool and methodology used can help public health officials plan future programs that will protect the significant investments made to eliminate canine rabies.

## Methods

### Overview

We analyzed four hypothetical scenarios involving the reintroduction of canine rabies into an area where canine rabies has been eliminated (Table 1). The reintroduction of canine rabies is defined as the re-establishment of onward transmission after either a human-aided importation, a natural incursion, or a host-shift event. Our scenarios account for varying levels of reintroduction risk over a 20-year period, defined by the intensity (number of dogs coming in and initiating chains of onward transmission) and the frequency (how often incursion events are happening). Each scenario can be considered as varying either expected risk of reintroduction or strength of the surveillance system. For example, a low-risk scenario is suitable for an island far from rabies endemic countries with less frequent incursion, or an area with a strong surveillance system that would catch most reintroductions.

**Table 1:** Four hypothetical scenarios of canine rabies reintroduction and the definition of successfully preventing sustained onward transmission. Scenario 1 represents the lowest risk, while scenario 4 corresponds to the highest risk of reintroduction. Scenarios 2 and 3 represent intermediate risks, which can be described as ‘high number of dogs reintroduced with low frequency’ and ‘low number of dogs reintroduced with high frequency,’ respectively.

Scenario	Intensity (number of dogs)	Frequency	Definition of successfully preventing sustained onward transmission
1	Single dog	Once at the beginning of year 1, over a 20-year period.	Rabies is eliminated (i.e., annual cases of rabid dogs decrease and stay below 1) within 2 years of reintroduction, then the area remains rabies-free.
2	10 dogs		
3	Single dog	Every 3 years, over a 20-year period (i.e., at the beginning of year 1, 4, 7, etc.)	Rabies is eliminated (i.e., annual cases of rabid dogs decrease and stay below 1) within 2 years of reintroduction, after every 3-year incursion.
4	10 dogs		

We used a modified version of RabiesEcon [13], which is a deterministic Susceptible-Exposed-Infected-Recovered (SEIR) mathematical model, to evaluate the potential impacts and cost-effectiveness of vaccination strategies to prevent the reintroduction of canine rabies. Detailed descriptions of the transmission models used in this paper are available in S1 Appendix. S2 Appendix contains a list of modifications made to RabiesEcon and a copy of the spreadsheet-based tool.

### Model inputs

To model the reintroduction scenarios, we used a hypothetical area of 100 km^2^ in which approximately half a million humans reside, with a human-to-susceptible dog ratio of 15:1. This is representative of a densely populated area, which is based on the average population density of urban areas in Asian and African countries [14]. The susceptible dog population represents free-roaming dogs with no immunity to rabies. We list in Table 2 other input variables, their default values, and sources.

**Table 2:** Demographic and epidemiologic data used in the analysis. The user can alter all the variables using the accompanying tool (S2 Appendix) for the target area of interest.

Input	Default Value	Reference
Size of the program area (km^2^)	100	Assumed
Human population	534,722	Demographia World Urban Areas [14]
Humans per km^2^	5,347	Derived
Human birth rate (per 1,000 population)	18.5	UN World Population Prospects [15]
Human life expectancy, years	72	WHO Global Health Observatory Data [16]
Human-to-Susceptible dog ratio	15:1	Knobel et al. [17]
Density of susceptible dogs	357 dogs/km^2^	Derived
Dog birth rate (per 1,000 dogs)	530	Hampson et al. [18]
Dog life expectancy, years	3.0	Zinsstag et al. [19]
Rabies R_0_ Dog-to-Dog	1.2	Hampson et al. [18]
Dog-Human transmission rate	0.0002054	Zinsstag et al. [19]
Percent of exposed humans receiving PEP	90%	Assumed
Rabies vaccine efficacy	95%	WHO Expert Consultation on Rabies [4]
Loss of dog vaccine immunity	0.0036/week	Based on a meta-analysis (S3 Appendix)

### Interventions

If there is a risk of reintroduction in a rabies-free area, it may be necessary to continue vaccinating portions of susceptible dog populations to prevent the re-establishment of onward transmission. We assumed that public health authorities would prefer to keep costs, and thus the percentage of dogs vaccinated as low as possible while remaining effective. Therefore, for each reintroduction scenario, we explored the minimum vaccination coverage required to prevent the re-establishment of canine rabies. We assumed that the vaccination campaign is targeted at the susceptible dog population, and is not aimed at the well-supervised dogs (confined, partially confined) or previously vaccinated free-roaming dogs. To compare the cost-effectiveness of various coverages, we also considered the WHO recommended level of 70% to eliminate rabies.

We assumed that an annual dog vaccination program would last for 10 weeks, and the efficacy of vaccine biologicals used is 95%, as per WHO guidelines (4). In this study, we assumed that there is a stable dog population and there are no additional interventions (such as sterilization, confinement or culling) other than mass dog vaccination and human post-exposure prophylaxis (PEP). In many endemic countries, only 30% to 60% of people bitten receive PEP [20, 21]. However, we assume that in a setting where canine rabies has been eliminated, the rabies program also has the capacity to ensure relatively high coverage of PEP compared to other endemic countries. Therefore, we assumed that PEP is provided to 90% of exposed people. We further assumed, based on evidence from Amparo et al. [22, 23], that 10 people are treated with PEP for each truly exposed case.

### Costs and cost-effectiveness

We included the following categories of costs for the intervention programs: cost of dog vaccination, human PEP, and costs associated with suspect rabies exposures (Table 3). As the majority of CRVV-endemic countries are in Africa and Asia, we adopted cost estimates from East Africa. All cost data are adjusted to 2018 USD, and we did not discount future costs or benefits. We used a governmental perspective (government-as-payer) and thus did not include any costs incurred by the patient such as a medical copay or time lost from work.

**Table 3:** Cost estimates used in the analysis. The user can alter any cost estimates in the table using the accompanying tool (S2 Appendix) for the target area of interest.

Category	Estimates	Reference
Cost of the dog vaccination program		
Vaccines	$0.69/unit	
Syringes & Needles	$0.12/unit	
Vaccination Certificates	$0.01/unit	
Dog marking	$0.02/unit	
Vaccine wastage percentage	10%	
Total direct medical costs	$0.91/unit	[24–27]
Workers at vaccination site	$28,198	
Transportation	$18,536	
Miscellaneous materials	$19,752	
Total indirect costs	$66,486	[24–27]
Average cost per dog vaccinated assuming 50% of target dog population is vaccinated	$4.65	Calculated
Cost of human PEP		
Cost per vaccine dose (material, overhead, vaccine)	$15.71	[17, 24]
Total doses required for PEP regimen	4	[4]
Average cost of Rabies Immune Globulin (RIG)	$143.65	[17, 28]
Proportion of PEP patients receiving RIG	7%	[17]
Average cost of PEP*	$72.91	Calculated
Cost of suspect exposure		
Lab test	$7.19	[17]
Bite investigation	$21.84	[29]

*We assumed the use of human rabies immunoglobin (HRIG) among 7% of PEP recipients. Using an alternative RIG (equine rabies immunoglobin; ERIG) would only marginally reduce the average cost of human PEP. For example, assuming $20 per ERIG [30], the average cost of PEP becomes $64.24.

To assess the cost-effectiveness of maintaining sufficient coverage over a 20-year period, we calculated the average cost per human death averted (i.e., cost-effectiveness ratio; CER) as follows:

$$CER=\frac{{Total\;program\;cost\;over\;20\;years}_{intervention}}{{Total\;human\;deaths}_{no\;intervention}-{Total\;human\;deaths}_{intervention}}.$$

### Sensitivity analyses

To determine the relative importance of model parameters, we varied each of the seven variables listed in Table 4. We first performed univariate sensitivity analyses by varying one variable at a time. With scenarios 3 and 4 where reintroductions occur every 3 years over a 20-year period, we varied the frequency of the incursion. To evaluate the scenario for rural areas or an area with a sparsely populated susceptible dog population, we varied the density of the susceptible dog population by changing the human-to-susceptible dog ratio to 1500:1. We also varied the dog birth rate and dog life expectancy. In addition, we assessed the scenario assuming a lower vaccine efficacy. Based on a meta-analysis on the rate of loss of vaccine immunity in mostly free-roaming dog populations with potential health issues (S3 Appendix), we examined the impact of a more rapid decrease in dog vaccine immunity. Assuming an exponential decay, we changed the rate to be 0.95% of vaccinated dogs losing vaccine immunity each week (i.e., 61% of vaccinated dogs remain immunized by the end of each year). We also examined the impact of various rates of dog-to-dog transmission by varying the basic reproduction number (R_0_) from 1.1 (stable, endemic populations) to 1.8 (epizootic events). This is the average (expected) number of new cases produced by a single infectious dog in a completely susceptible population.

**Table 4:** List of variables, default value and their uncertainty range used in the sensitivity analyses.

Variable	Default value	Alternative values
Frequency of reintroduction, scenarios 3 and 4	Every 3 years	Every 10 years
Density of susceptible dog population (human-to-susceptible dog ratio)	357 dogs/km^2^ (15:1)	3.6 dogs/km^2^ (1500:1)
Dog birth rate	530 per 1,000 dogs	300 per 1,000 dogs 670 per 1,000 dogs
Dog life expectancy	3 years	2 and 5 years
Dog vaccine efficacy	95%	80%
Loss of dog vaccine immunity	0.0036/week	0.0095/week
Basic reproduction number (R_0_)	1.2	1.1 to 1.8

We also conducted multivariate sensitivity analyses to study the combined impact and the uncertainties around our assumption. We explored a list of settings by varying multiple variables at a time as described in Table 5.

**Table 5:** Settings for the multivariate sensitivity analyses.

**Setting 1.** Dog rabies vaccine	
1-1.	Vaccine efficacy 95%; Loss of vaccine immunity 0.0036/week
1-2.	Vaccine efficacy 80%; Loss of vaccine immunity 0.0095/week
**Setting 2.** Dog demographics	
2–1.	Density of susceptible dogs 3.6/km^2^; Dog birth rate 300 per 1,000; Life expectancy 5 years
2–2.	Density of susceptible dogs 356/km^2^; Dog birth rate 670 per 1,000; Life expectancy 2 years
**Setting 3.** Best and worst-case	
3–1.	Density of susceptible dogs 3.6/km^2^; Dog birth rate 300 per 1,000; Life expectancy 5 years; Vaccine efficacy 95%; Loss of vaccine immunity 0.0036/week; R_0_ = 1.1
3–2.	Density of susceptible dogs 356/km^2^; Dog birth rate 670 per 1,000; Life expectancy 2 years; Vaccine efficacy 80%; Loss of vaccine immunity 0.0095/week; R_0_ = 1.8

In addition, we considered a set of additional scenarios to account for varying levels of detection probabilities. We varied the number of dogs reintroduced from 0.01 to 100 dogs for each scenario. A lower number of reintroduced dogs can be considered as a high detection rate, by allowing only a fraction of reintroduced dogs to initiate the chain of onward transmission while others were assumed to be identified, captured and quarantined.

## Results

### Base case analysis

We first explored the minimum vaccination coverage required to prevent the re-establishment of dog rabies from each reintroduction scenario. With scenario 1 (single dog incursion, once at the beginning of year 1), annually vaccinating 38% of susceptible dogs will end the chain of onward transmission within the first 2 years of reintroduction. With scenario 4 where 10 dogs are reintroduced every 3 years, 56% of the dogs must be annually vaccinated to prevent re-establishment of CRVV in the dog population (Table 6).

**Table 6:** Minimum vaccination coverage required to prevent re-establishment of dog rabies from each reintroduction scenario.

Scenario	Intensity (number of dogs)	Frequency	Minimum vaccination coverage required
1	Single dog	Once at the beginning of year 1, over a 20-year period.	38% 49%
2	10 dogs		
3	Single dog	Every 3 years, over a 20-year period (i.e., at the beginning of year 1, 4, 7, etc.)	47% 56%
4	10 dogs		

Next, we compared the cost-effectiveness of various vaccination strategies. The strategies included no intervention, PEP only, vaccinating at a minimum coverage required, and at 70% vaccination coverage.

Across all scenarios, maintaining dog vaccination at the minimum coverage required yields the lowest cost per rabies-related human death averted. We presented the results from the lowest risk (scenario 1) and the highest risk (scenario 4) settings in Tables 7 and 8. Results from the intermediate-risk settings (scenarios 2 and 3) and the cost breakdown of various vaccination strategies are available in S4 and S5 Appendices.

**Table 7:** Cumulative health and economic impacts of various vaccination strategies with scenario 1 (single dog incursion, once at the beginning of year 1).

	Total dog rabies cases*	Average annual incidence rate per 1,000 dogs	Total human deaths	Average annual incidence rate per 100,000 humans	Total program cost	Average cost per human death averted	CRVV re-established
No intervention	32,910	53.02	5,461	49.09	$0	-	Y
No vaccination, PEP only	32,910	53.02	792	7.12	$17,835,146	$3,820	Y
Vaccinate 38% dogs with PEP	6	0.009	0	0.002	$1,405,380	$257	N
Vaccinate 70% dogs with PEP	2	0.002	0	0.001	$2,583,198	$473	N

**Table 8:** Cumulative health and economic impacts of various vaccination strategies with scenario 4 (10 dogs reintroduced every 3 years).

	Total dog rabies cases*	Average annual incidence rate per 1,000 dogs	Total human deaths	Average annual incidence rate per 100,000 humans	Total program cost	Average cost per human death averted	CRVV re-established
No intervention	33,705	54.78	5,582	50.36	$0	-	Y
No vaccination, PEP only	33,705	54.78	810	7.30	$18,240,581	$3,822	Y
Vaccinate 56% dogs with PEP	214	0.48	7	0.06	$2,217,899	$398	N
Vaccinate 70% dogs with PEP	113	0.34	4	0.04	$2,679,740	$480	N

*Total dog rabies cases over 20 years do not include cases reintroduced to the area, from an importation, incursion or host-shift event.

With scenario 1, maintaining dog vaccination at 38% was most cost-effective among other vaccination options, with a cost-effectiveness ratio of $257 per human death averted (Table 7). If PEP is provided to 90% of exposed humans without any mass dog vaccination campaign, it costs $3,820 to prevent one rabies-related human death. In addition, canine rabies becomes re-established thus incurring significant costs to re-eliminate. When there is a risk of reintroduction, discontinuing both dog vaccination and human PEP entirely would result in approximately 33,000 rabid dog cases and 5,500 human deaths over 20 years (Table 7). With scenario 4, maintaining 56% coverage (minimum vaccination coverage required) had the lowest cost-effectiveness ratio of $398 per human death averted (Table 8).

### Sensitivity analyses

#### Univariate sensitivity analyses

**Fig 1:**
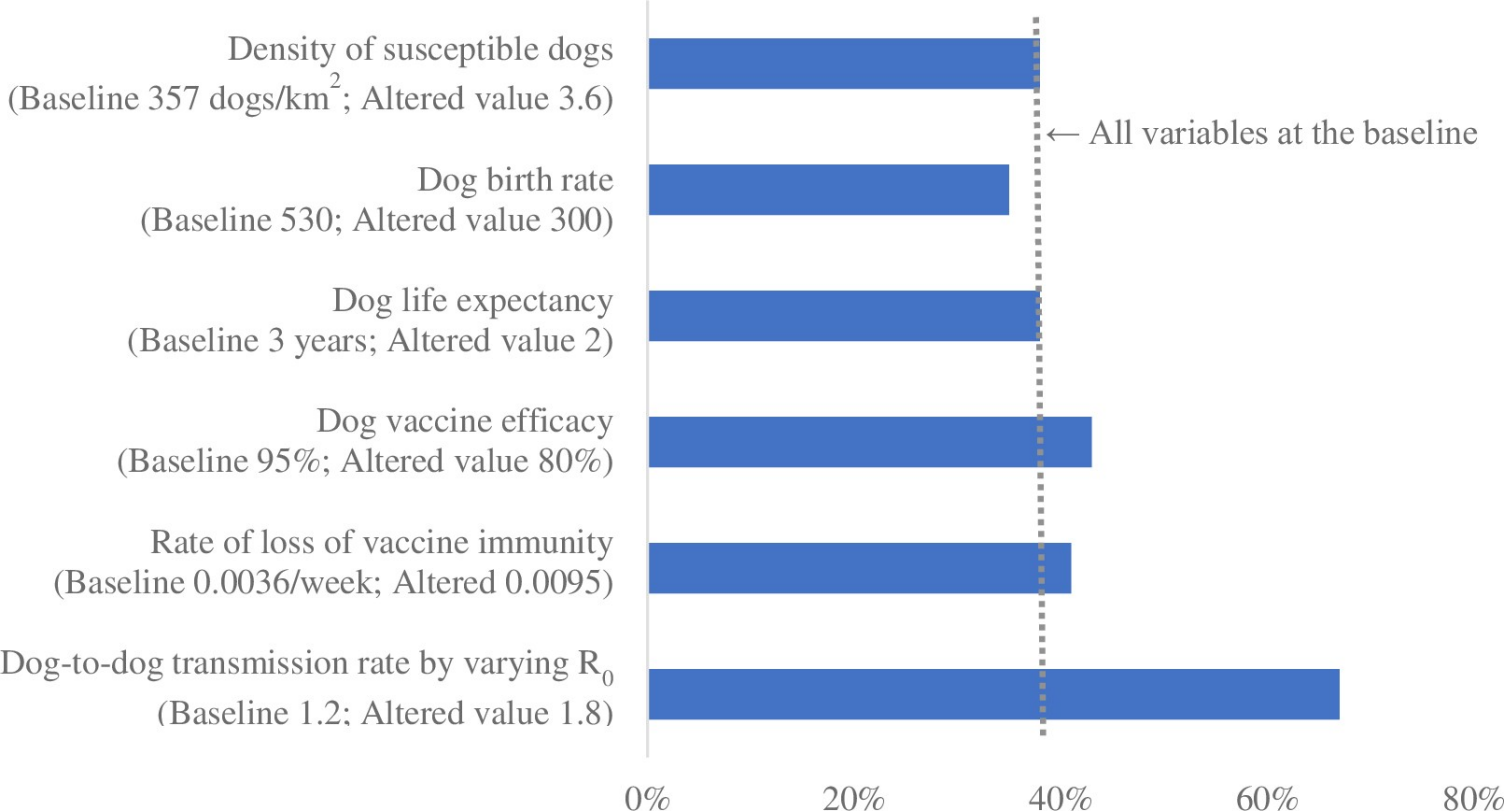
The relative impact of changing each input variable on the minimum vaccination coverage required to prevent re-establishment of rabies; reintroduction scenario 1 (single dog incursion, once at the beginning of year 1).

**Fig 2:**
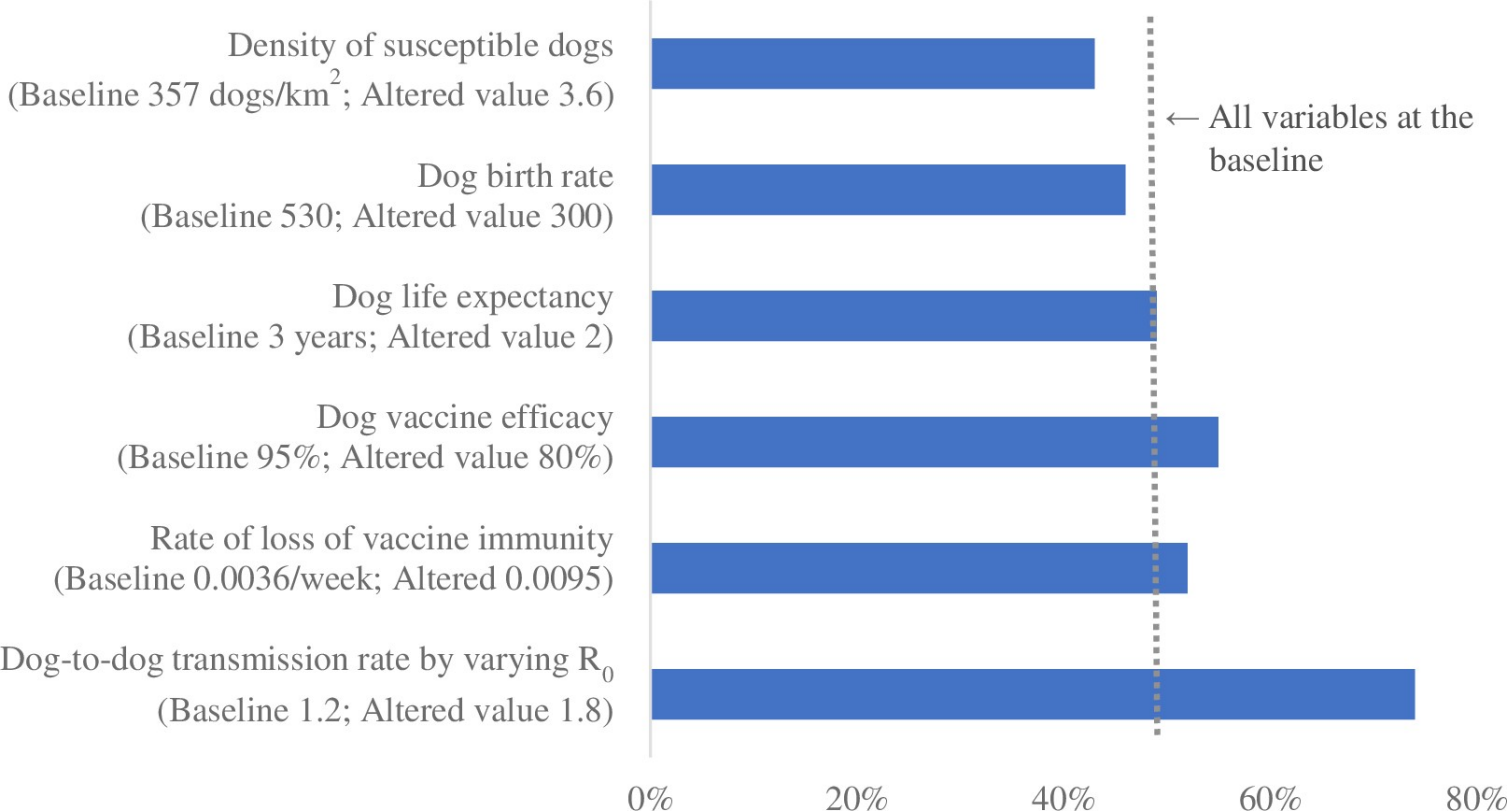
The relative impact of changing each input variable on the minimum vaccination coverage required to prevent re-establishment of rabies; reintroduction scenario 2 (10 dogs reintroduced, once at the beginning of year 1).

**Fig 3:**
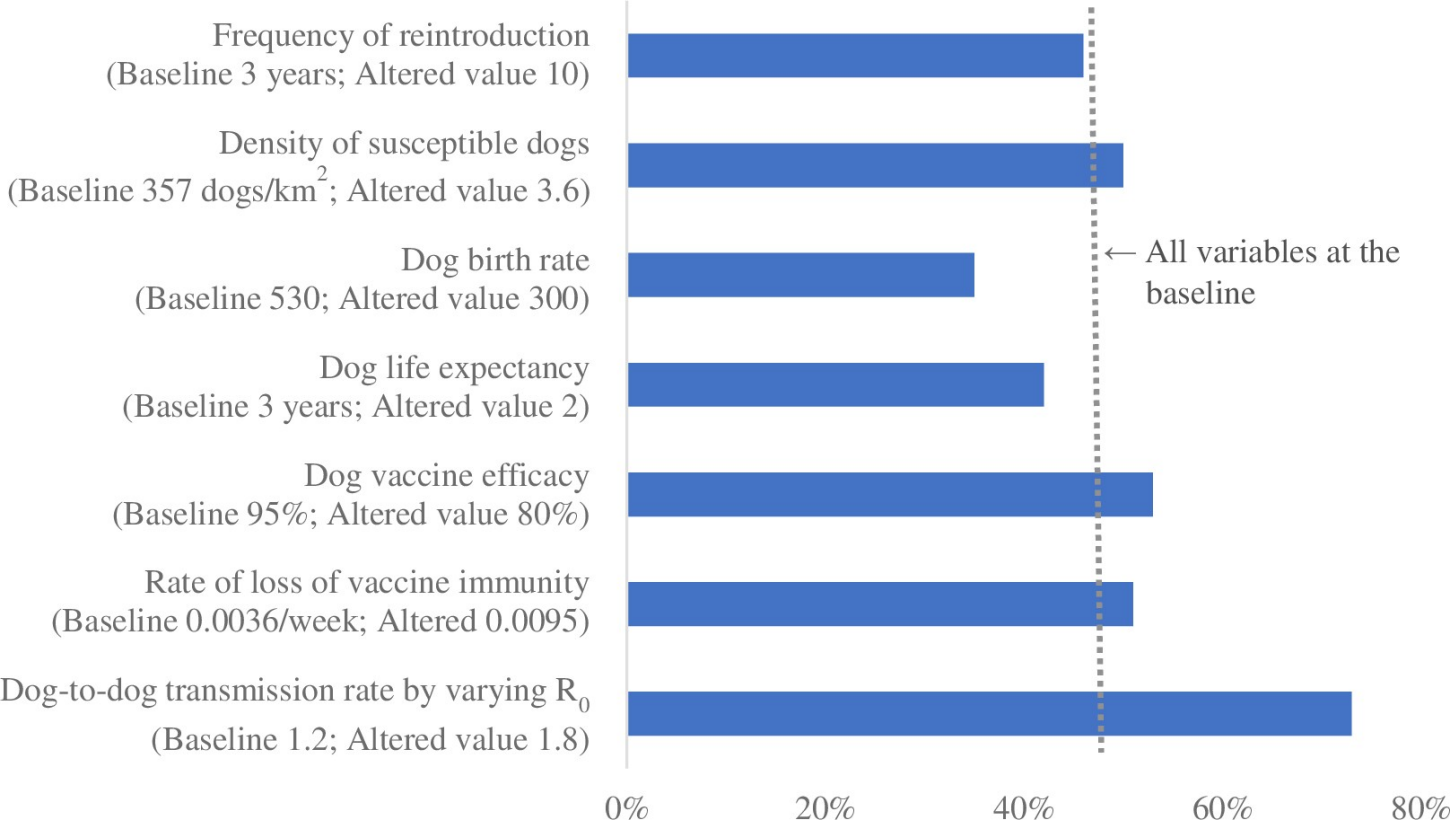
The relative impact of changing each input variable on the minimum vaccination coverage required to prevent re-establishment of rabies; reintroduction scenario 3 (single dog reintroduced every 3 years).

**Fig 4:**
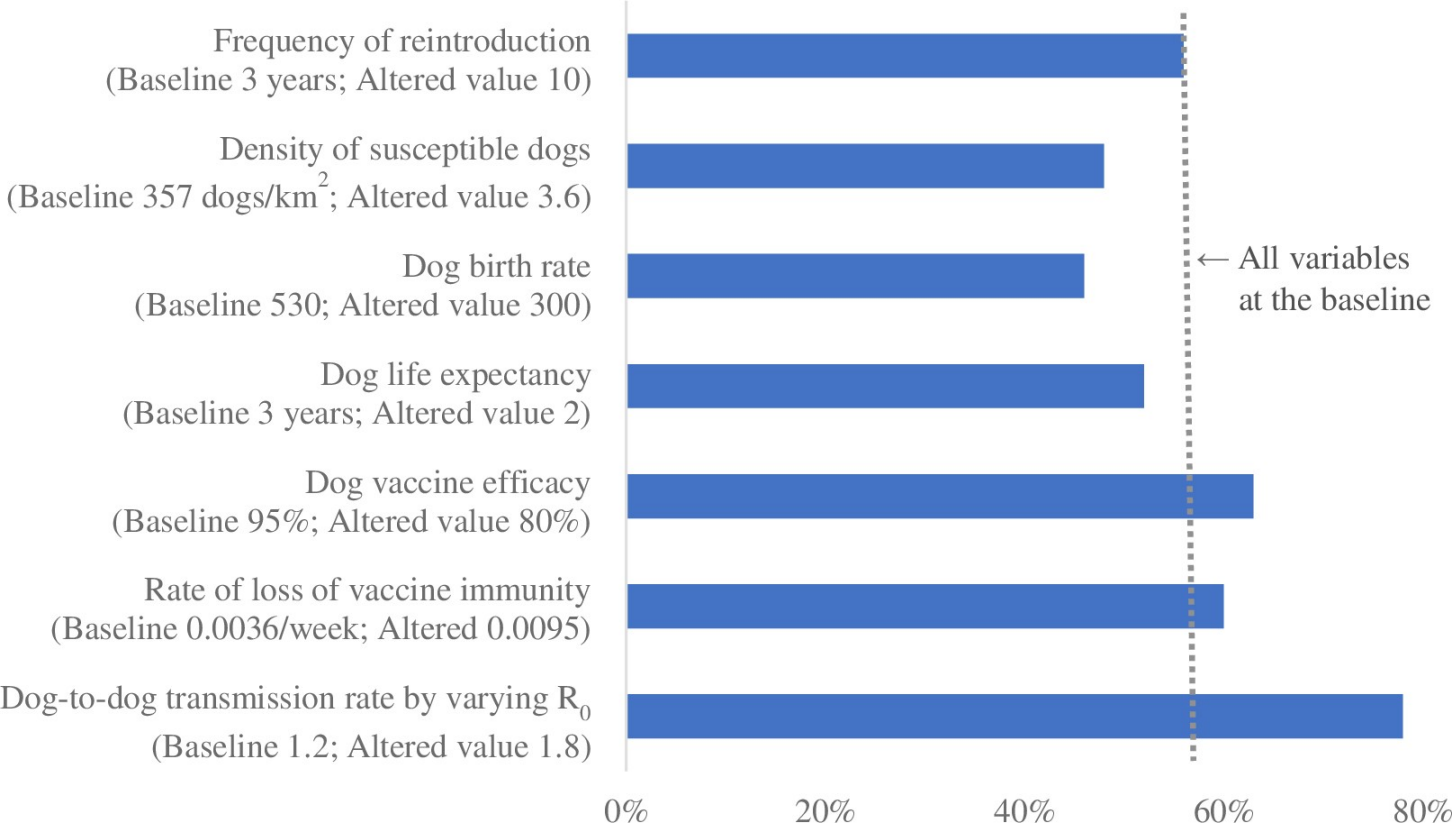
The relative impact of changing each input variable on the minimum vaccination coverage required to prevent re-establishment of rabies; reintroduction scenario 4 (10 dogs reintroduced every 3 years).

Figs 1–4 summarize the relative impact of varying each input variable on the outcome variable. We varied one variable at a time and displayed the result from the alternative value. If multiple alternative values were assessed (e.g., R_0_), we presented the value that moved the outcome variable the most.

Across all scenarios, the minimum vaccination coverage required was most sensitive to the dog-to-dog transmission rate (modeled by varying R_0_), followed by the vaccine efficacy and the rate of loss of vaccine immunity. The density of the susceptible dog population played an important role when a large number of dogs were reintroduced (scenarios 2 and 4). Dog birth rate and life expectancy had a substantial impact on the outcome variable when there were frequent incursions (scenarios 3 and 4). Lowering the frequency of reintroduction from every 3 years to every 10 had a minimal impact.

**Fig 5:**
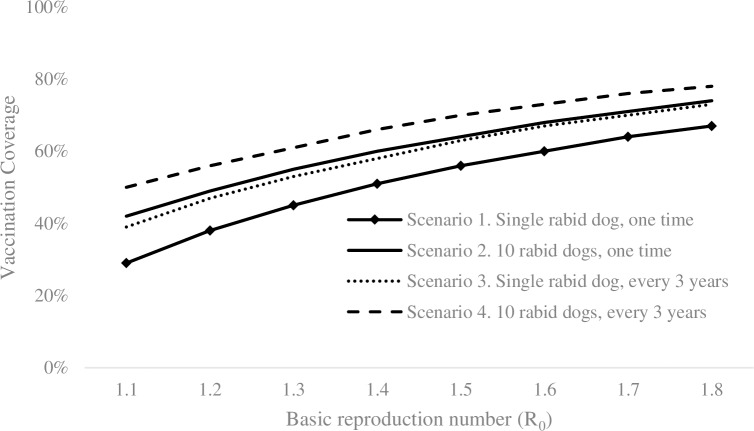
Minimum vaccination coverage required to prevent re-establishment of rabies, when varying dog-dog transmission rate (which is modeled by varying R_0_ from 1.1 to 1.8) for each reintroduction scenario.

Fig 5 further explores the impact of the dog-to-dog transmission rate on the minimum vaccination coverage required for each of the 4 reintroduction scenarios. As one rabid dog generates more infected cases (i.e., dog-to-dog transmission rate increases), higher vaccination coverage is required to prevent re-establishment of canine rabies. For example, with scenario 1, 29% of dogs need to be annually vaccinated to prevent re-establishment of dog rabies when R_0_ = 1.1, whereas the rate increases to 67% when R_0_ is 1.8. Note that the area needs to vaccinate at least 70% of dogs when the dog-to-dog transmission rate is relatively high (R_0_ > 1.8) even with the lowest risk of reintroduction.

#### Multivariate sensitivity analyses

As shown in Table 9, varying vaccine efficacy and the rate of loss of vaccine immunity had a similar impact across all 4 scenarios. In other words, when the rabies vaccine had lower efficacy and a higher rate of loss of immunity, the area needs to vaccinate an additional 8% to 11% of the susceptible dog population to prevent the re-establishment of rabies. On the other hand, dog demographics had a more substantial impact when there was a high risk of reintroduction. If demographic and epidemiologic variables listed in Table 5 were controlled to represent a best-case scenario (susceptible dogs are sparsely populated with low birth rate and high life expectancy; vaccine has high efficacy with longer duration; low dog-to-dog transmission rate), the minimum vaccination coverage could be lowered to 27% to 37% regardless of the level of reintroduction risk (Setting 3–1).

**Table 9:** Minimum vaccination coverage required to prevent re-establishment of dog rabies when varying a set of variables as defined in Table 5.

	Reintroduction Scenarios			
	Scenario 1	Scenario 2	Scenario 3	Scenario 4
1–1. Vaccine has 95% efficacy with longer duration	38%	49%	47%	56%
1–2. Vaccine has 80% efficacy with shorter duration	46%	59%	57%	67%
2–1. Dogs are sparsely populated with low birth rate, longer life expectancy	35%	39%	44%	39%
2–2. Dogs are densely populated with high birth rate, shorter life expectancy	39%	50%	49%	58%
3–1. Best case based on vaccine, dog demographics and transmission rate	27%	32%	37%	32%
3–3. Worst case based on vaccine, dog demographics and transmission rate	80%	86%	85%	89%

#### Varying levels of detection probability

When varying the number of reintroduced dogs that initiated the chain of onward transmission, we found that the minimum vaccination coverage varied as much as 30% depending on the detection probability (29% to 59% with a single incursion; 33% to 65% with multiple incursions every 3 years) as shown in Table 10. Note that even with a high detection rate, the area needs to vaccinate 29% to 33% of susceptible dogs to prevent re-establishment of rabies (Scenarios 1-A and 3-A).

**Table 10:** Minimum vaccination coverage required to prevent re-establishment of dog rabies when varying the number of dogs reintroduced (i.e., detection probability) for each scenario.

Scenario	Intensity (number of dogs)	Frequency	Minimum vaccination coverage required
1-A	0.01 dog	Once at the beginning of year 1, over a 20-year period	29%
1-B	0.1 dog		31%
1	Single dog		38%
2	10 dogs		49%
2-A	100 dogs		59%
3-A	0.01 dog	Every 3 years, over a 20-year period	33%
3-B	0.1 dog		38%
3	Single dog		47%
4	10 dogs		56%
4-A	100 dogs		65%

## Discussion

In this paper, we explored one of the post-elimination rabies control strategies, a preventive vaccination. We consider rabies reintroduction scenarios with varying levels of risk of rabies re-introduction, the density of susceptible dog population, dog birth rate, dog life expectancy, vaccine efficacy, rate of loss of vaccine immunity, and the basic reproduction number (R_0_). When there is a low risk of reintroduction (single dog incursion, once at the beginning of year 1), maintaining mass dog vaccination with 38% coverage among susceptible dogs is sufficient to prevent rabies from re-establishing endemicity. The average cost per human death averted with 38% vaccination coverage is $257 over a 20-year period, whereas the average cost is 1.8 times higher with 70% vaccination coverage. Vaccinating more dogs than necessary may not be the optimal use of government funds. It also can have potentially undesirable health impacts such as dogs being exposed to unnecessary adverse reactions, injuries during capture, aggressive dog-on-dog interactions at static-point vaccination clinics, or vaccinators at risk of traumatic bite events. In comparison, when PEP is provided without any dog vaccination campaign, it costs $3,820 to prevent one rabies-related human death. It is not a single cost-effective intervention, nor will it eliminate human deaths unless dog vaccination is included in the program. On the other hand, discontinuing vaccination and human PEP entirely would result in approximately 33,000 rabid dog cases and 5,500 human deaths over 20 years when there is a risk of reintroduction and poor ability to detect incursion events. With a high risk of reintroduction (10 dogs reintroduced every 3 years), the area needs to maintain 56% coverage among the susceptible dog population. Countries should also assess their surveillance capacity and consider alternatives to preventive vaccination, such as border control, active and passive surveillance, or ring vaccination when cases occur. These may be more cost-effective when correctly implemented.

As the minimum vaccination coverage required to prevent re-establishment of rabies was most sensitive to the dog-to-dog transmission rate, it is important to understand the range of the basic reproduction number (R_0_) for the target area. R_0_ can vary greatly, from extremely low values (as low as 1.05) with stable, endemic populations [18] to extremely high values (as high as 2.42) during epizootic events [31]. Multiple researchers have modeled R_0_, but there is still a lack of agreement on whether rabies is a density or frequency-transmitted disease as both mechanisms are plausible for rabies [32]. If it is modeled as density-dependent then contact rates and therefore R_0_ would increase as dog density increases, but if it is modeled as frequency-dependent then contact rates and R_0_ would remain constant unless affected by other variables such as age structure or local extinctions.

Measuring R_0_ in a field setting is difficult to do in a reliable and timely manner for rabies control. Without strong surveillance data, rabies control programs may only be able to quantify their local transmission dynamics as high transmission risk (R_0_ ≥ 1.5), moderate (1.2 ≤ R_0_ < 1.5) and low (R_0_ < 1.2). As shown in Fig 5, dog population dynamics that would result in low transmission risk may require as low as 29% vaccination coverage, whereas dog populations where varying factors favor high transmission risk would require greater than 67% coverage. Program managers should be aware of the importance of this designation on the estimated vaccination coverage requirements; under-vaccination could place human and animal lives at risk and jeopardize a rabies-free status.

The preventive vaccination coverage was also sensitive to dog rabies vaccine (vaccine efficacy, rate of loss of vaccine immunity) and dog demographics (density, birth rate, life expectancy). While the vaccine-related variables had a consistent impact across various levels of reintroduction risk, dog demographics had a more substantial impact when there was a high risk of reintroduction. We also found that even with a relatively high detection rate, the area needs to vaccinate portions of susceptible dog population (29% to 33%) to prevent the re-establishment of rabies. Therefore, it may be worth considering an ongoing, preventive vaccination targeted at a high-risk area (e.g., area of immediate risk or a bordering area to a highly endemic region).

The risk of reintroduction is dependent on numerous factors, including proximity to an infected area, border security to control the movement of dog populations, surveillance capacity, and reactive or preventative disease control actions. Countries that are geographically isolated from endemic areas would likely be considered low-risk for reintroduction, and an event occurring once over a 20-year period may be reflective of this situation (scenario 1). In this setting, vaccinating 38% of susceptible dogs is likely to suffice to prevent the re-establishment of dog rabies within the first 2 years of reintroduction. The coverage could be further reduced if the area can ensure sustained, strong surveillance capacity with a high detection rate. If there is a much higher risk of reintroduction (e.g., 10 dogs reintroduced every 3 years), the area may need to vaccinate at least 56% of susceptible dogs each year. This may be more reflective of situations in Peru (bordering endemic Bolivia) and Malaysia (bordering endemic Indonesia and Thailand). In both of these country settings, the endemic area was adjacent to the free area and there was no standard or adequately enforced restriction of dog movement across the border. In these high-risk settings, the area would need an additional $0.8M in a 20-year period to prevent re-establishment of rabies, compared to the low-risk setting. If neighbors and large areas around the country were rabies-free, then perhaps further reduction or total discontinuation of mass dog vaccination would be possible. Therefore, there are financial benefits to implementing importation laws that could prevent rabies reintroduction or to help neighbors achieve canine rabies elimination. Cross-border sharing of rabies surveillance data is also essential as this will inform and influence the preventive vaccination coverage of the bordering area.

One of the limitations of the deterministic model we adopted in this paper is that there is no built-in uncertainty. Therefore, we performed extensive sensitivity analyses and assessed which variable (or combination of variables) had the most substantial impact on the outcome. In addition, we assumed that the population is well-mixed, i.e., all interactions (between susceptible to infectious dogs, and susceptible humans to infectious dogs) occur homogeneously, at constant rates, and continuously over time. This is a simple representation of the transmission dynamics, and it may not be suitable for a specific context such as the spatial distribution of rabies over large regions. However, our simple model can be useful in settings where data is limited [33], or when the intervention is aimed at a small, targeted area.

The reintroduction scenario includes a two-fold purpose; determining the minimum vaccination coverage required to prevent re-establishment of rabies and informing policymakers on the cost of continuing or discontinuing vaccination programs post-elimination. We acknowledge that the reintroduction scenario presented here is relatively aggressive by assuming there is an inevitable reintroduction. Therefore, our study provides a lower limit of cost-effectiveness ratio that the decision-makers could leverage.

Rabies is a viral illness with a high case fatality rate that can result in a heavy disease burden. However, when rabies gets under control, available funds and control efforts are often lowered or canceled altogether. Our study suggests that there is a need for thorough planning beyond elimination. When considering a preventive vaccination strategy in an area with a potential risk of rabies reintroduction, neither continuing mass vaccination at 70% nor discontinuing altogether is practical. This assessment aims to help countries that are at risk of reintroduction decide how to implement policies for preventive vaccination that best suit their epidemiological and economic situation, especially in areas with poor surveillance capacity.

### Disclaimer

The findings and conclusions in this report are those of the authors and do not necessarily represent the official position of the Centers for Disease Control and Prevention.

## Supporting information

S1 Appendix Description of the transmission models used in the paper. (DOCX)

S2 Appendix List of modifications made to RabiesEcon. (DOCX)

S3 Appendix Meta-analysis comparing the rate of loss of vaccine immunity. (DOCX)

S4 Appendix Results from the cost-effectiveness analysis of reintroduction scenarios 2 and 3. (DOCX)

S5 Appendix Cost breakdown of various vaccination strategies. (DOCX)

S6 Appendix Accompanying tool–RabiesEcon_Reintro. (XLSX)
