# Supplementary material for: Determining the post-elimination level of vaccination needed to prevent re-establishment of dog rabies
Source: PLoS Negl Trop Dis. 2019 Dec 2;13(12):e0007869. doi: 10.1371/journal.pntd.0007869 (PMC6907870; doi:10.1371/journal.pntd.0007869)
Supplement: S1 Appendix — (DOCX) [file pntd.0007869.s001.docx]

**Determining the post-elimination level of vaccination needed to prevent re-establishment of dog rabies**

Seonghye Jeon^1*^, Julie Cleaton^2^, Martin I. Meltzer^1^, Emily B. Kahn^1^, Emily G. Pieracci^2^, Jesse D. Blanton^2^ and Ryan Wallace^2^

**Appendix S1.** Description of the transmission models used in the paper

Mathematical models are used to understand the disease transmission dynamics, the impact of intervention strategies and the uncertainties around the outcome. There are various modeling approaches including compartmental or individual-based, deterministic or stochastic, and homogeneous or heterogeneous-mixing patterns [1]. In this paper, we choose one of the simple models, a deterministic SEIR compartmental model with homogeneous mixing. This approach requires fewer assumptions and therefore is useful in settings where data (from which we can estimate the parameters) is scarce [2]. In addition, the model and the accompanying tool is designed in a way that the user can alter any model parameters including demographic, geographic, or epidemiologic data to better fit the target area of interest.

The following equations describe the SEIR transmission models used in this paper, adapted from Borse et al. [3] and Zinsstag et al. [4]. For a glossary of parameters used in these equations, see Supplemental Table S1.

**Equations used to define Dog-to-Dog transmission model**

$\frac{{dS}_{d}}{dt}=b_{d}N_{d}+\lambda_{d}R_{d}+(\sigma_{d}{(1-r}_{d})E_{d})-m_{d}S_{d}-\beta_{d}S_{d}I_{d}-{\gamma N}_{d}S_{d}-v_{d}\alpha_{d}S_{d}$

$$\frac{{dE}_{d}}{dt}=\beta_{d}S_{d}I_{d}-m_{d}E_{d}-{\gamma N}_{d}E_{d}- (\sigma_{d}{(1-r}_{d})E_{d})-v_{d}\alpha_{d}E_{d}-\sigma_{d}r_{d}E_{d}$$

$$\frac{{dI}_{d}}{dt}= \sigma_{d}r_{d}E_{d} -m_{d}I_{d}-{\gamma N}_{d}I_{d}-\mu_{d}I_{d}$$

$$\frac{{dR}_{d}}{dt}= (v_{d}{\alpha_{d}(S_{d}+E}_{d})) -m_{d}R_{d}-{\gamma N}_{d}R_{d}-\lambda_{d}R_{d}$$

$$\gamma=\frac{{(b}_{d}-m_{d})}{K}$$

**Equations used to define Dog-to-Human transmission model**

$$\frac{{dS}_{h}}{dt}={(b}_{h}(S_{h}+E_{h}+R_{h}))+\lambda_{h}R_{h}+E_{h}\left( \frac{P2\left( 1-P6 \right)}{i_{head}}+\frac{P3\left( 1-P7 \right)}{i_{arm}}+\frac{P4\left( 1-P8 \right)}{i_{trunc}}+\frac{P5\left( 1-P9 \right)}{i_{leg}} \right){-m}_{h}S_{h}{-\beta}_{dh}S_{h}I_{d}$$

$$\frac{{dE}_{h}}{dt}=\beta_{dh}S_{h}I_{d}{-m}_{h}E_{h}-{P10\nu_{h}E}_{h}\left( \frac{P2P6}{i_{head}}+\frac{P3P7}{i_{arm}}+\frac{P4P8}{i_{trunk}}+\frac{P5P9}{i_{leg}} \right)-{(1-P10\nu_{h})E}_{h}\left( \frac{P2P6}{i_{head}}+\frac{P3P7}{i_{arm}}+\frac{P4P8}{i_{trunk}}+\frac{P5P9}{i_{leg}} \right) -E_{h}\left( \frac{P2\left( 1-P6 \right)}{i_{head}}+\frac{P3\left( 1-P7 \right)}{i_{arm}}+\frac{P4\left( 1-P8 \right)}{i_{trunk}}+\frac{P5\left( 1-P9 \right)}{i_{leg}} \right)$$

$$\frac{{dI}_{h}}{dt}=(1-P10\nu_{h})E_{h}\left( \frac{P2P6}{i_{head}}+\frac{P3P7}{i_{arm}}+\frac{P4P8}{i_{trunk}}+\frac{P5P9}{i_{leg}} \right){-m}_{h}I_{h}-\mu_{h}I_{h}$$

$$\frac{dR_{h}}{dt}=P10\nu_{h}E_{h}\left( \frac{P2P6}{i_{head}}+\frac{P3P7}{i_{arm}}+\frac{P4P8}{i_{trunk}}+\frac{P5P9}{i_{leg}} \right)-m_{h}R_{h}-\lambda_{h}R_{h}$$

Immunized

Dogs

Susceptible

Dogs

Exposed

Dogs

Rabid

Dogs

Vaccination

Loss of
Immunity

Infection

Abortive
Exposure

Mortality

Mortality

Mortality

Clinical Disease

Birth

Mortality

Vaccination

Rabies

Mortality

Immunized

Humans

Susceptible Humans

Exposed

Humans

Rabid

Humans

Loss of
Immunity

Infection

Abortive
Exposure

Mortality

Mortality

Mortality

Clinical Disease

Birth

Mortality

PEP Vaccination

Rabies

Mortality

**Supplemental Figure S1.** Diagram summarizing rabies transmission models for dog-to-dog and dog-to-human transmissions; adapted from Zinsstag et al. [4].

**Supplemental Table S1.** Glossary of parameters

| **Symbol** | **Variable** | | **Default value** | **Reference** | **User can alter the value** |
| --- | --- | --- | --- | --- | --- |
| *T* | | Time | 1 week |  | N |
| *S_d_* | | Susceptible dog population per km^2^ | Calculated (dogs/km^2^) |  | Y |
| *E_d_* | | Exposed dog population per km^2^ | Calculated (dogs/km^2^) |  | N |
| *I_d_* | | Infectious (rabid) dog population per km^2^ | Calculated (dogs/km^2^) |  | N |
| *R_d_* | | Immune dog population per km^2^ | Calculated (dogs/km^2^) |  | N |
| *N_d_* | | Total dog population per km^2^ | Calculated (dogs/km^2^) | User Input | Y |
| *b_d_* | | Dog birth rate | 530/1000/52 (dogs/week) | [5] | Y |
| *λ_d_* | | Loss of rabies vaccine immunity in dogs | 0.0036 (week^-1^) | Appendix S3 | Y |
| *i_d_* | | Dog rabies incubation period in weeks | 6.27 (week) | [6] | Y |
| *σ_d_* | | Inverse of average incubation period | *1/ i_d_* | Calculated | N |
| *r_d_* | | Risk of clinical outcome | 0.45 | [4] | Y |
| *dog_life* | | Dog life expectancy | 3 (years) | [4] | Y |
| *m_d_* | | Dog death rate | 1/*dsog_life*/52 (week^-1^) | Calculated | N |
| *β_d_* | | Dog-dog transmission coefficient (inverse of time between dog contacts) | $R_{0}\frac{\left( \sigma_{d}+m_{d} \right)\left( m_{d}+\mu_{d} \right)}{\sigma_{d}r_{d}S_{d}}$ (km^2^/dogs/week) | Derived from [4] | Y |
| *γ* | | Dog density dependent mortality | $\frac{b_{d}-m_{d}}{K}$ (km^2^/dogs/week) | Calculated | N |
| *K* | | Mean carrying capacity | $1.05\cdot N_{d}\left( 1+\frac{1}{ln(\# total dog)} \right)$  (dogs/km^2^) | [3] | N |
| *v_d_* | | Dog vaccine efficacy | 0.95 | [7] | Y |
| *vaccine* | | Vaccination coverage |  | User Input | Y |
| *α_d_* | | Dog vaccination rate  (weeks 1-10) | $-\frac{1}{10}ln(1-vaccine)$ (week^-1^) | Calculated | N |
|  | | Dog vaccination rate  (weeks 11+) | 0 (week^-1^) | Assumed | N |
| *infective* | | Dog rabies infective period | 5 (days) | [3] | Y |
| *μ_d_* | | Rabid mortality rate | $7\cdot\frac{1}{infective}$ (week^-1^) | Calculated | N |
| *S_h_* | | Susceptible human population per km^2^ | Calculated (humans/ km^2^) |  | N |
| *E_h_* | | Exposed human population per km^2^ | Calculated (humans/ km^2^) |  | N |
| *I_h_* | | Rabid human population per km^2^ | Calculated (humans/ km^2^) |  | N |
| *R_h_* | | Immune human population per km^2^ | Calculated (humans/ km^2^) |  | N |
| *N_h_* | | Total human population per km^2^ | Calculated (humans/ km^2^) | User Input | Y |
| *b_h_* | | Human birth rate | 18.5/1000/52 (week^-1^) | [8] | Y |
| *λ_h_* | | Human loss of vaccination immunity rate | 0 (week^-1^) | [3] | N |
| *human_life* | | Human life expectancy | 72 (years) | [9] | Y |
| *m_h_* | | Human mortality rate | 1/*human_life*/52 (week^-1^) | Calculated | N |
| *β_dh_* | | Dog human transmission rate | 0.0002054 (km^2^/dogs/week) | [4] | Y |
| *P10* | | Human post exposure prophylactic (PEP) vaccination rate | 90% (week^-1^) | Assumed | Y |
| *v_h_* | | Human vaccine efficacy | 0.95 | [7] | Y |
| *P2* | | Probability of a bite to the head | 0.070 | [10] | Y |
| *P3* | | Probability of a bite to the arm | 0.384 | [10] | Y |
| *P4* | | Probability of a bite to the trunk | 0.060 | [10] | Y |
| *P5* | | Probability of a bite to the leg | 0.486 | [10] | Y |
| *P6* | | Probability of developing rabies after a bite to the head | 0.450 | [10] | Y |
| *P7* | | Probability of developing rabies after a bite to the arm | 0.275 | [10] | Y |
| *P8* | | Probability of developing rabies after a bite to the trunk | 0.050 | [10] | Y |
| *P9* | | Probability of developing rabies after a bite to the leg | 0.050 | [10] | Y |
| *i_head_* | | Human incubation period after bite to the head | 3.14 (weeks) | [11] | Y |
| *i_arm_* | | Human incubation period after bite to the arm | 8.57 (weeks) | [11] | Y |
| *i_trunk_* | | Human incubation period after bite to the trunk | 6.43 (weeks) | [11] | Y |
| *i_leg_* | | Human incubation period after bite to the leg | 10.71 (weeks) | [11] | Y |
| *infective_h* | | Average infective period for humans | 7 (days) | [12] | Y |
| *μ_h_* | | Inverse of average infective period, rabid human mortality rate | $7\cdot\frac{1}{infective\_h}$ (week^-1^) | Calculated | N |

**REFERENCES**

1. Muellner U, Fournié G, Muellner P, Ahlstrom C, Pfeiffer DU. epidemix—An interactive multi-model application for teaching and visualizing infectious disease transmission. Epidemics. 2018;23:49-54.

2. Panjeti VG, Real LA. Mathematical Models for Rabies. In: Jackson AC, editor. Advances in Virus Research2011. p. 377-95.

3. Borse RH, Atkins CY, Ganbhir M, Undurraga EA, Blanton JD, Kahn EB, et al. Cost-effectiveness of dog rabies vaccination programs in East Africa. PLOS Neglected Tropical Diseases. 2018;23(12):e0006490.

4. Zinsstag J, Dürr S, Penny MA, Mindekem R, Roth F, González SM, et al. Transmission dynamics and economics of rabies control in dogs and humans in an African city. Proceedings of the National Academy of Science of the USA. 2009;106(35):14996-5001.

5. Hampson K, Dushoff J, Cleaveland S, Haydon DT, Kaare M, Packer C, et al. Transmission dynamics and prospects for the elimination of canine rabies. PLOS Biology. 2009;7(3):462-71.

6. Foggin CM. Rabies and rabies-related viruses in Zimbabwe: Historical, virological and ecological aspects (Doctoral thesis): University of Zimbabwe; 1988.

7. WHO Expert Consultation on Rabies: Second report. 2013.

8. UN World Population Prospects, 2017 Revision: United Nations Population Division; [cited 2019 March 1]. Available from: <https://population.un.org/wpp/>.

9. WHO Global Health Observatory Data 2016 [cited 2019 March 1]. Available from: <https://www.who.int/gho/en/>.

10. Cleaveland S, Fevre EM, Kaare M, Coleman PG. Estimating human rabies mortality in the United Republic of Tanzania from dog bite injuries. Bulletin of the World Health Organization. 2002;80(4):304-10.

11. Sudarshan M, Madhusudana S, Mahendra B, Rao N, Ashwath Narayana D, Abdul Rahman S, et al. Assessing the burden of human rabies in India: results of a national multi-center epidemiological survey. International Journal of Infectious Diseases. 2007;11(1):29-35.

12. WHO Recommended Surveillance Standards (Second edition) 2007 [cited 2019 July 1]. Available from: <https://www.who.int/csr/resources/publications/surveillance/whocdscsrisr992.pdf>.
