# Supplementary material for: Determining the post-elimination level of vaccination needed to prevent re-establishment of dog rabies
Source: PLoS Negl Trop Dis. 2019 Dec 2;13(12):e0007869. doi: 10.1371/journal.pntd.0007869 (PMC6907870; doi:10.1371/journal.pntd.0007869)
Supplement: S2 Appendix — (DOCX) [file pntd.0007869.s002.docx]

**Determining the post-elimination level of vaccination needed to prevent re-establishment of dog rabies**

Seonghye Jeon^1*^, Julie Cleaton^2^, Martin I. Meltzer^1^, Emily B. Kahn^1^, Emily G. Pieracci^2^, Jesse D. Blanton^2^ and Ryan Wallace^2^

**Appendix S2.** List of modifications made to RabiesEcon

In this paper, we used a modified version of RabiesEcon [1]. Below are some key modifications made to the original model. The modified tool is available in S6 Appendix.

- The modified version uses a single study area rather than encompassing separate data fields for urban and rural areas.
- The modified version does not include the initial 10,000 weeks of the burn-in period built to arrive at a steady-state (i.e., endemic state). Instead, it allows the reintroduction of canine rabies into a rabies free area with varying risk (intensity and frequency of reintroduction).
- The original version of the model assumes an initial ‘endemic’ steady state, with the number of exposed humans during the 1^st^ year being used to calculate associated costs (suspect exposure costs, PEP costs) for the following years. However, the modified version calculates the projected number of human exposures and associated costs for each year.
- The modified version includes an inflation factor to account for people receiving PEP when they are not truly exposed to canine rabies. As a default, we assumed that 10 people are treated with PEP for each truly exposed case.
- Instead of using the rate of loss of vaccine immunity from Akakpo et al. [2], we performed a meta-analysis and used the pooled rate from the random-effects model. Details can be found in Appendix S3.
- The user can alter the ‘basic reproduction number (R_0_)’ directly instead of entering the ‘number of bites per dog to another dog’ and the ‘risk of clinical outcome per bite’ separately.
- The modified tool displays the cumulative health impacts of each vaccination strategy on the main page.

**REFERENCES**

1. Borse RH, Atkins CY, Gambhir M, Undurraga EA, Blanton JD, Kahn EB, et al. Cost-effectiveness of dog rabies vaccination programs in East Africa. PLOS Neglected Tropical Diseases. 2018;23(12):e0006490.

2. Akakpo A, Mbou G, Bornarel P, Sarradin P, Bada A. Serologic response in dogs after a mass primary antirabies vaccination (inactivated vaccine) at Pikine Dakar (Senegal). Dakar Medical. 1993;38(2):123-8.
