# Supplementary material for: Determining the post-elimination level of vaccination needed to prevent re-establishment of dog rabies
Source: PLoS Negl Trop Dis. 2019 Dec 2;13(12):e0007869. doi: 10.1371/journal.pntd.0007869 (PMC6907870; doi:10.1371/journal.pntd.0007869)
Supplement: S3 Appendix — (DOCX) [file pntd.0007869.s003.docx]

**Determining the post-elimination level of vaccination needed to prevent re-establishment of dog rabies**

Seonghye Jeon^1*^, Julie Cleaton^2^, Martin I. Meltzer^1^, Emily B. Kahn^1^, Emily G. Pieracci^2^, Jesse D. Blanton^2^ and Ryan Wallace^2^

**Appendix S3.** Meta-analysis comparing the rate of loss of vaccine immunity

We identified 4 studies that reported the seropositive rate 1-year post vaccination against dog rabies (Supplemental Table S2). We then performed a meta-analysis to obtain the pooled rate (Supplemental Figure S2). As the heterogeneity across studies were statistically significant, we used the random-effects model. The pooled rate (0.83) and the lower bound (0.61) were used for the base case and the sensitivity analysis. Assuming an exponential decay, this is equivalent to 0.36% and 0.95% of vaccinated dogs losing vaccine immunity each week, respectively.

**Supplemental Table S2.**  List of studies used in the meta-analysis

| **Source** | **Year** | **Country** | **Vaccine** | **Number of dogs** | **Serology Test** | **Titer Cutoff** | **Seropositive** |
| --- | --- | --- | --- | --- | --- | --- | --- |
| Chomel et al. (1) | 1987 | Peru | Unknown | 198 | RFFIT | 0.5 IU/mL | 97% |
| Sage et al. (2) | 1993 | US (Alaska) | Rabdomun | 21 | RFFIT | 0.5 IU/mL | 67% |
| Bahloul et al. (3) | 2006 | Tunisia | Rabisin | 62 | RFFIT | 0.5 IU/mL | 73% |
| Zinsstag et al. (4) | 2017 | Chad | Rabisin | 58 | Unknown | 0.5 IU/mL | 76% |


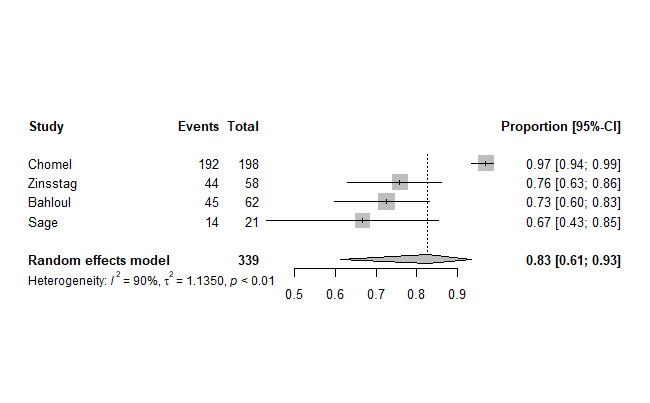
 **Supplemental Figure S2.** Forest plot from the meta-analysis conducted to obtain pooled rate of loss of vaccine immunity. The *I^2^* statistics describes the percentage of variation across studies that is due to heterogeneity rather than random chance, and *τ^2^* is the estimated between-study variance. There is statistically significant heterogeneity across studies at the significance level 0.05, as the p-value is less than 0.01.

**REFERENCES**

1. Chomel B, Chappuis G, Bullon F, Cardenas E, Beublain TD, Maufrais MC, et al. Serological results of a dog vaccination campaign against rabies in Peru. Revue Scientifique et Technique-Office International. 1987;6:97-113.

2. Sage G, Khawplod P, Wilde H, Lobaugh C, Hemachudha T, Tepsumethanon W, et al. Immune response to rabies vaccine in Alaskan dogs: failure to achieve a consistently protective antibody response. Transactions of The Royal Society of Tropical Medicine and Hygiene. 1993;87(5):593-5.

3. Bahloul C, Taieb D, Kaabi B, Diouani MF, Hadjahmed SB, Chtourou Y, et al. Comparative evaluation of specific ELISA and RFFIT antibody assays in the assessment of dog immunity against rabies. Epidemiology and Infection. 2005;133(4):749-57.

4. Zinsstag J, Lechenne M, Laager M, Mindekem R, Naïssengar S, Oussiguéré A, et al. Vaccination of dogs in an African city interrupts rabies transmission and reduces human exposure. Science Translational Medicine. 2017;9(421).
