# Supplementary material for: Determining the post-elimination level of vaccination needed to prevent re-establishment of dog rabies
Source: PLoS Negl Trop Dis. 2019 Dec 2;13(12):e0007869. doi: 10.1371/journal.pntd.0007869 (PMC6907870; doi:10.1371/journal.pntd.0007869)
Supplement: S4 Appendix — (DOCX) [file pntd.0007869.s004.docx]

**Determining the post-elimination level of vaccination needed to prevent re-establishment of dog rabies**

Seonghye Jeon^1*^, Julie Cleaton^2^, Martin I. Meltzer^1^, Emily B. Kahn^1^, Emily G. Pieracci^2^, Jesse D. Blanton^2^ and Ryan Wallace^2^

**Appendix S4.** Results from the cost-effectiveness analysis of reintroduction scenarios 2 and 3

**Supplemental Table S3.** Cumulative health and economic impacts of various vaccination strategies with scenario 2 (10 dogs reintroduced, once at the beginning of year 1).

|  | Total dog rabies cases | Average annual incidence rate per 1,000 dogs | Total human deaths | Average annual incidence rate per 100,000 humans | Total program cost | Average cost per human death averted | CRVV  re-established |
| --- | --- | --- | --- | --- | --- | --- | --- |
| No intervention | 33,488 | 54.24 | 5,538 | 49.96 | $0 | - | Y |
| No vaccination, PEP only | 33,488 | 54.24 | 803 | 7.24 | $18,091,832 | $3,821 | Y |
| Vaccinate 49% dogs with PEP | 33 | 0.05 | 1 | 0.01 | $1,829,338 | $330 | N |
| Vaccinate 70% dogs with PEP | 15 | 0.02 | 1 | 0.01 | $2,594,672 | $469 | N |

**Supplemental Table S4.** Cumulative health and economic impacts of various vaccination strategies with scenario 3 (single dog reintroduced every 3 years).

|  | Total dog rabies cases | Average annual incidence rate per 1,000 dogs | Total human deaths | Average annual incidence rate per 100,000 humans | Total program cost | Average cost per human death averted | CRVV  re-established |
| --- | --- | --- | --- | --- | --- | --- | --- |
| No intervention | 32,923 | 53.06 | 5,464 | 49.12 | $0 | - | Y |
| No vaccination, PEP only | 32,923 | 53.06 | 793 | 7.12 | $17,844,806 | $3,820 | Y |
| Vaccinate 47% dogs with PEP | 37 | 0.07 | 1 | 0.01 | $1,757,459 | $333 | N |
| Vaccinate 70% dogs with PEP | 11 | 0.03 | 0 | 0.004 | $2,591,711 | $474 | N |
