# Supplementary material for: Determining the post-elimination level of vaccination needed to prevent re-establishment of dog rabies
Source: PLoS Negl Trop Dis. 2019 Dec 2;13(12):e0007869. doi: 10.1371/journal.pntd.0007869 (PMC6907870; doi:10.1371/journal.pntd.0007869)
Supplement: S5 Appendix — (DOCX) [file pntd.0007869.s005.docx]

**Determining the post-elimination level of vaccination needed to prevent re-establishment of dog rabies**

Seonghye Jeon^1*^, Julie Cleaton^2^, Martin I. Meltzer^1^, Emily B. Kahn^1^, Emily G. Pieracci^2^, Jesse D. Blanton^2^ and Ryan Wallace^2^

**Appendix S5.** Cost breakdown of various vaccination strategies

**Supplemental Table S5.** Cost breakdown of various vaccination strategies with scenario 1 (single dog incursion, once at the beginning of year 1).

|  | Dog vaccination costs, 20 years | PEP costs,  20 years | Suspect exposure costs^*^, 20 years | Total program cost, 20 years |
| --- | --- | --- | --- | --- |
| No intervention | - | - | - | $0 |
| No vaccination, PEP only | $0 | $17,530,033 | $305,113 | $17,835,146 |
| Vaccinate 38% dogs with PEP | $1,401,554 | $3,760 | $65 | $1,405,380 |
| Vaccinate 70% dogs with PEP | $2,581,897 | $1,279 | $22 | $2,583,198 |

^*^Suspect exposure costs include costs from lab test and bite investigation.

**Supplemental Table S6.** Cost breakdown of various vaccination strategies with scenario 2 (10 dogs reintroduced, once at the beginning of year 1).

|  | Dog vaccination costs, 20 years | PEP costs,  20 years | Suspect exposure costs, 20 years | Total program cost, 20 years |
| --- | --- | --- | --- | --- |
| No intervention | - | - | - | $0 |
| No vaccination, PEP only | $0 | $17,782,327 | $309,504 | $18,091,832 |
| Vaccinate 49% dogs with PEP | $1,806,937 | $22,017 | $383 | $1,829,338 |
| Vaccinate 70% dogs with PEP | $2,581,662 | $12,787 | $223 | $2,594,672 |

**Supplemental Table S7.** Cost breakdown of various vaccination strategies with scenario 3 (single dog reintroduced every 3 years).

|  | Dog vaccination costs, 20 years | PEP costs,  20 years | Suspect exposure costs, 20 years | Total program cost, 20 years |
| --- | --- | --- | --- | --- |
| No intervention | - | - | - | $0 |
| No vaccination, PEP only | $0 | $17,539,527 | $305,278 | $17,844,806 |
| Vaccinate 47% dogs with PEP | $1,733,265 | $23,781 | $414 | $1,757,459 |
| Vaccinate 70% dogs with PEP | $2,581,780 | $9,761 | $170 | $2,591,711 |

**Supplemental Table S8.** Cost breakdown of various vaccination strategies with scenario 4 (10 dogs reintroduced every 3 years)

|  | Dog vaccination costs, 20 years | PEP costs,  20 years | Suspect exposure costs, 20 years | Total program cost, 20 years |
| --- | --- | --- | --- | --- |
| No intervention | - | - | - | $0 |
| No vaccination, PEP only | $0 | $17,928,532 | $312,049 | $18,240,581 |
| Vaccinate 56% dogs with PEP | $2,063,374 | $151,881 | $2,644 | $2,217,899 |
| Vaccinate 70% dogs with PEP | $2,580,495 | $97,548 | $1,698 | $2,679,740 |
